# Variations of physical properties, bioactive phytochemicals, antioxidant capacities and PPO activities of cantaloupe melon (*Cucumis melo* L.) slices subjected to different drying methods

**DOI:** 10.3389/fnut.2025.1548271

**Published:** 2025-05-19

**Authors:** Xin Gao, Junyan Yang, Haojie Wang, Jiancai Mao, Lipeng Wu, Junhua Li, Xuhui Wang

**Affiliations:** ^1^Hami Melon Research Center, Xinjiang Academy of Agricultural Sciences, Urumqi, China; ^2^Institute of Nuclear Technology and Biotechnology, Xinjiang Academy of Agricultural Sciences, Urumqi, China

**Keywords:** cantaloupe melon, drying methods, bioactive compounds, antioxidant activities, PPO activity

## Abstract

In order to prolong the market life and maintain the health benefits of cantaloupe melon (*Cucumis melo* L.), the present study compared the influences of three commonly used methods in drying cantaloupe melon slices. Drying methods applied included microwave drying (MD), freeze drying (FD), and hot air drying at 40°C (HD40), 50°C (HD50), 60°C (HD60), and 70°C (HD70). Physical changes such as moisture, color, volume shrinkage ratio, hardness, and rehydration ratio under different drying methods were firstly evaluated. Besides, content of free amino acids, soluble sugar, total phenolics, flavonoids, and some individual bioactive compounds such as ascorbic acid, β-carotene, rutin, gallic acid, chlorogenic acid, and caffeic acid were measured. In addition, *in vitro* antioxidant activities evaluated by DPPH, FRAP and ABTS methods and polyphenol oxidase activities were also determined. On the whole, FD was the best method in maintaining physical changes, content of phytochemicals and antioxidant activities. MD was better than HD in drying cantaloupe melon slices. Of the HD treatments, HD60 was better than others. The findings of the present study can provide the scientific basis for future large-scale production of dried cantaloupe melon slices.

## 1 Introduction

Melon (*Cucumis melo* L.), is an important tropical fruit that belongs to the Cucurbitaceae family. Melon is widely distributed in temperate, subtropical, and tropical areas of the world (1). Major melon producing countries include China, Turkey, the United States, Spain and Iran (2). According to the data of FAO, the annual planting area of melons was 1.72 million hectares in the world, with the output of over 40 million tons and the export value of 2.4 billion US dollars every year (2). Among so many different kinds of melons, cantaloupe melon has high economic value worldwide due to its sweet and juicy taste, pleasing flavor, and nutritional value (3). Cantaloupe melon is rich in vitamin C (ascorbic acid), β-carotene, minerals and many other antioxidant active ingredients such as flavonoids and phenolic acids (4, 5). These functional ingredients possess many health benefits such as anti-oxidant, anti-inflammation, and preventing or repairing cellular damage in the body (6). It has been demonstrated that consumption of cantaloupe melon possesses therapeutic and preventive roles against a number of chronic diseases such as inflammation, degenerative diseases and some kinds of cancers (7). In addition, cantaloupe melon was reported to have stomachic, diuretic, and vermifuge effect (7).

However, fresh cantaloupe melon usually contains high water content of about 90% (8). Complex physiological and biochemical reactions still exist in cantaloupe melon after harvest, which can cause the reduction of nutritional ingredients and bioactive components (8). As the characteristics of high moisture content and perishability of fresh cantaloupe melon, it has a short shelf life of only about 15 days (9). Besides, cantaloupe melon is easily damaged during sale, resulting in great economic losses (10). In order to extend the shelf life and maintain the health benefits of cantaloupe melon, appropriate processing technology must be used to decrease the water content and water activity of cantaloupe. Dehydration is one of the useful and important methods to preserve the beneficial properties of fruits in the post-harvest processes, as drying can not only inhibit the physiological and biochemical reactions, but also limits the growth of microorganisms (11–13).

Different methods of drying have been applied for plant-based food with different unique characteristics. The parameters under traditional drying methods such as shading drying and sun drying cannot be controlled, thus it is difficult to guarantee the quality, efficacy and consistency of the final products (14). In comparison, hot air drying or oven-drying has many advantages against sun drying, such as controllable drying conditions and reduced microbial contamination, resulting in the higher quality and more uniform products (14). Microwave drying can also be an alternative method with shorter drying time, thus leading to less adverse effect on nutritional values of dried fruits (15). As for freeze drying, fruits are frozen at first, and then it is dehydrated through sublimation. Because there is no liquid water and the temperature under this process is very low, most metamorphic and microbial reactions are inhibited, which makes the final dried products have excellent qualities (15). Different drying process may have different influences on the appearance, aroma components, chemical compositions and biological activities of fruits (16). For example, microwave vacuum drying was better than hot air drying for lemon slices (17). Freeze drying could influence the bioactivity of finger citron (18). Some phenolics of strawberry was significantly increased by freeze-drying (19). Freeze drying resulted in higher content of bioactive components, and antioxidative activity than air drying in green legumes (20).

A large-scale production of consistent dry cantaloupe melon slices requires a comprehensive understanding of the factors that influence the quality and bioactivity of cantaloupe during the drying process. Therefore, determination of the factors that contribute to the quality and bioactivity of cantaloupe melon slices is of great importance. However, until now, few detailed studies have been conducted on the factors that influence the quality and no guidelines have been established for drying cantaloupe melon slices. Thus, with the aim of determining the best drying method for cantaloupe melon slices, three different methods widely reported in the literature were used to dry cantaloupe melon. The methods included hot air drying (HD), microwave drying (MD), and freeze drying (FD). HD was conducted at 40°C (HD40), 50°C (HD50), 60°C (HD60), and 70°C (HD70), respectively. This study assessed the effects of different drying methods on color, volume shrinkage ratio, hardness, rehydration ratio, and content of free amino acids, soluble sugar, total phenolics, flavonoids, and some individual bioactive compounds. Besides, the antioxidant activities and under different drying conditions were also evaluated using DPPH, ABTS, and FRAP methods. Lastly, the polyphenol oxidase activities of cantaloupe melon slices were compared.

## 2 Materials and methods

### 2.1 Reagents and chemicals

Ascorbic acid, β-carotene, rutin, gallic acid, chlorogenic acid, caffeic acid, anhydrous glucose, anhydrous sodium sulfate and ninhydrin were purchased from Yuanye Biotechnology Co. (Shanghai, China) with the purity of over 98%. Citric acid, sodium chloride, hydrochloric acid, phenol and sulfuric acid were purchased from Sinopharm Group Chemical reagent Co., LTD (Shanghai, China) with the purity of analytical grade. Folin–Ciocalteu reagent, 2,2-diphenyl-1-picrylhydrazyl (DPPH), 2,2′-azinobis (3-ethylbenzothiazoline-6-sulfonic acid) (ABTS), 2,4,6-tri(2-pyridyl)-1,3,5-triazine (TPTZ), and leucine and were got from Sigma Chemical Co. (St. Louis, MO, United States) with the purity of over 95%. Acetonitrile, methanol and tetrahydrofuran of HPLC grade were obtained from ACS (Houston, TX, United States). All other chemicals or reagents were of analytical grade.

### 2.2 Cantaloupe melon samples

The fresh cantaloupe melon samples (variety Xizhoumi No.25) used for this study were purchased from the local market (Urumqi, Xinjiang, China) and stored at 4°C before drying. Mature cantaloupe melon that was with the same maturity and without any mechanical injuries were chosen. Each cantaloupe melon used had uniformed size, weight, and moisture with the average length, diameter, weight, and initial moisture of 26.5 ± 2.5 cm, 17.5 ± 2.5 cm, 3.15 ± 0.25 kg, and 90.5 ± 0.15%, respectively.

### 2.3 Drying methods

#### 2.3.1 Pre-drying treatment

Before the drying process, the fresh cantaloupe melon was washed, peeled and deseeded. Edible parts of the melon were sliced into 30 × 50 × 8 mm thin slices. And then the slices were immersed into a mixed water solution containing citric acid (0.15%) and sodium chloride (0.15%) for color protection. After 20 min, the slices were taken out and rinsed, and the surface water was removed by an absorbent paper.

An equal amount of fresh samples was dried by the following methods: microwave drying (MD), freeze drying (FD), hot air drying at 40°C (HD40), hot air drying at 50°C (HD50), hot air drying at 60°C (HD60), or hot air drying at 70°C (HD70). Fresh samples were processed by the respective drying methods described below until they were dried to a constant weight (±0.005 g).

#### 2.3.2 Microwave drying

Pre-treated cantaloupe melon slices were dried in a microwave oven (NJ07-3; Nanjing Jiequan Microwave Apparatus Co. Ltd., Nanjing, China) with a power level of 5 Watts/g. The actual power was divided by the weight of cantaloupe melon slices.

#### 2.3.3 Freeze drying

Pre-treated cantaloupe melon slices were put into a freezer at −80°C for 24 h, and then freeze-dried in a freeze-dryer (Shanghai Huxi Experimental Equipment Co., Ltd) under 20 Pa absolute pressure. The temperature of the cold trap and heating plate was set at about −58°C and 25°C, respectively.

#### 2.3.4 Hot air drying

Pre-treated cantaloupe melon slices were spread evenly on the salver and put into the drying chamber of the hot air dryer (Shanghai Yiheng Experimental Equipment Co., Ltd). The drying temperature was set at 40°C (HD40), 50°C (HD50), 60°C (HD60), and 70°C (HD70), respectively, with an air velocity of 2 m/s.

### 2.4 Determination of the physical properties

#### 2.4.1 Measurement of moisture

About 10 g samples were dried for 12 ± 0.5 h in an electric constant temperature drying oven (Shanghai Yiheng Experimental Equipment Co., Ltd) at 105°C, and the water content (%) was gravimetrically determined.

#### 2.4.2 Measurement of color difference

A colorimeter (Hangzhou CHNSpec Technology CO., LTD) was used to measure the color value of samples. The blank of the colorimeter was used as a standard. The measurements of one sample were replicated for 10 times, and the positions were randomly selected. Value L* represents the degree of darkness-lightness, value a* is the degree of greenness-redness, while value b* is the degree of blueness-yellowness. ΔE representing the total color difference was calculated using Equation 1.


(1) $$\mathrm{\Delta E}=\sqrt{{\left(L_{1}^{*}-L_{0}^{*}\right)}^{2}+{\left(a_{1}^{*}-a_{0}^{*}\right)}^{2}+{\left(b_{1}^{*}-b_{0}^{*}\right)}^{2}}$$

Where L∗_1_, a∗_1_, and b∗_1_ represent the values of fresh sample of melon and L∗_0_, a∗_0_, and b∗_0_ represent the values of the dried samples.

#### 2.4.3 Measurement of volume shrinkage ratio

The shrinkage ratio of cantaloupe melon slices was determined by the displacement method reported previously (21). The glass beads (0.1 mm, USA Scientific Inc., United States) were used as the replacement medium. The shrinkage ratio of dry cantaloupe melon slices was calculated using the following Equation 2.


(2) $$\text{Volume shrinkage ratio}\;(\%)=\frac{V1-V2}{V1}\times 100\;$$

Where V_1_ and V_2_ are the volume (cm^3^) of the fresh and dry cantaloupe melon slices, respectively.

#### 2.4.4 Measurement of hardness

The hardness of the cantaloupe melon slices, defined as the maximum force of the force deformation curve, was measured by a texture analyzer (CT3, Brookfield Ltd., MA, United States) (8). The dried cantaloupe melon slices were spread in a flat container with the thickness of 2 cm. A speed of 0.5 mm/s, distance of 0.8 cm, and trigger of 10 g was applied directly to the surface of cantaloupe melon slices until they were cracked.

#### 2.4.5 Measurement of rehydration ratio

The dried cantaloupe melon slices were weighted and soaked into water at room temperature. After soaked for 5 min, the samples were picked out and the surface water was removed using an absorbent paper. Afterwards, the dried samples were weighed and soaked in water again. These steps were repeated until the weight of the sample became stable (8). The rehydration ratio was calculated using the following Equation 3.


(3) $$\text{Rehydration ratio}\;(\%)=\frac{W1-W2}{W2}\times 100$$

Where W_1_ and W_2_ are the weight of the dry cantaloupe melon slices after and before rehydration (g), respectively.

### 2.5 Quantification of total sugar content and total free amino acids content

#### 2.5.1 Total sugar content

The total sugar content (TSC) of cantaloupe melon was measured based on the phenol-sulfuric acid method (22). About 1 g of cantaloupe melon samples were grinded thoroughly and put into a 250 mL flask. And then 50 mL of distilled water and 15 mL of concentrated hydrochloric acid were added and hydrolyzed with a boiling water bath for 3–4 h. Subsequently, the reaction mixture was filtered and the filtrate was adjusted to 200 mL by distilled water. Afterwards, 1 mL of the solution was pipetted and added into a glass tube and the volume was adjusted to 2 mL with distilled water. And then, 1 mL of phenol solution (5%, w: v) was added into the solution, followed by adding 5 mL of concentrated sulfuric acid. The mixture was rested for 10 min and then put into a water bath at 30°C for another 20 min. The absorbance at 490 nm was determined by a spectrophotometer (BioTek Instruments, Winooski, VT, United States). The total sugar content was determined by the standard curve of anhydrous glucose. Results were expressed as mg per gram of dry weight (mg/g DW).

#### 2.5.2 Total free amino acid content

The free amino acid content (TAC) of cantaloupe melon was measured by ninhydrin colorimetry (22). About 1 g of cantaloupe melon samples were homogenized with 10 mL of distilled water. The mixture was then put into a boiling water bath for 15 min prior to filtration. The filtrate was then collected into a beaker. Afterwards, 1 mL of phosphate buffer with the pH of 8.4, 1 mL of ninhydrin solution (1.2%, w:w) and 3 mL of distilled water was added into 1 mL of the filtrate. Subsequently, the mixture was boiled in a water bath for 15 min. And then the volume was adjusted to 25 mL. The absorbance at 570 nm was determined using a spectrophotometer (BioTek Instruments, Winooski, VT, United States). The total free amino acids content was calculated using the standard curve of leucine. Results were expressed as mg per gram of dry weight (mg/g DW).

### 2.6 Quantification of total phenolics content, total flavonoids content and total carotenoids content

#### 2.6.1 Sample extraction of carotenoids

Total carotenoid content (TCC) was measured as previously reported (23). About 2 g of cantaloupe melon samples were homogenized with 10 mL of cooled ethanol. And then 10 mL of hexane were added. Afterwards, the mixture was centrifuged at 5,000 g and 4°C for 10 min and the hexane layer was stored in dark. The residues were re-extracted by the same procedure until the residual solid became colorless. All the hexane extracts were mixed together and were saponified by adding 5 mL of methanolic (10%) potassium hydroxide solution in dark for about 2 h at room temperature. Afterwards, it was mixed with 50 mL of NaCl solution (10%) and the upper hexane layer was separated. Subsequently, the hexane layer was washed by deionized water until the pH of the rinse was neutral. Lastly, the hexane layer was dehydrated by anhydrous sodium sulfate.

#### 2.6.2 Quantification of total carotenoid content

The hexane extract of the cantaloupe melon samples was determined at the absorbance 450 nm using a spectrophotometer (BioTek Instruments, Winooski, VT, United States). The total carotenoid content was calculated according to the extinction coefficient (*ε*) of β-carotene in hexane (OD_450,_ ε^1%^_1cm_ = 2,500). Results were expressed as mg per 100 gram of dry weight (mg/100 g DW).

#### 2.6.3 Sample extraction for phenolics, flavonoids and antioxidant activity

About 2 g of cantaloupe melon samplewas homogenized with 30 mL of 80% aqueous ethanol, and sonicated for 30 min. After extracted for 24 h. the mixture was filtered. Subsequently, the filtrate was concentrated and diluted to 10 mL by the same extracting solvent. It was stored at −20°C for further analysis of phenolics, flavonoids and antioxidant activity.

#### 2.6.4 Quantification of total phenolics content

Total phenolics content (TPC) was measured using Folin–Ciocalteu reagent (24). Briefly, 100 μL of Folin–Ciocalteu reagent was added to 500 μL of the extract. 5 min later, 7 mL of 3% aqueous sodium carbonate solution was added into the mixture. Afterwards, it was placed into a water bath at 37°C in dark for 90 min. Subsequently, the absorbance at 765 nm was measured using spectrophotometer (BioTek instruments, Winooski, VT, United States). The TPC was calculated using the standard curve of gallic acid (GA). Results were shown as mg gallic acid equivalents per 100 gram of dry weight (mg GAE/100 g DW).

#### 2.6.5 Quantification of total flavonoids content

Total flavonoids content (TFC) was determined according to the method described by Liu et al. (25). 300 μL of 5% NaNO_2_ was added into 2 mL of diluted solutions of the extract. After 5 min, 300 μL of 10% AlCl_3_·6H_2_O was added. After 6 min, 2 mL of 4% NaOH was added. After 15 min, 5.4 mL of water was added to the mixture. Finally, the absorbance at 510 nm was measured by spectrophotometer (BioTek Instruments, Winooski, VT, United States). The TFC was calculated using the standard curve of rutin (RUT). Results were shown as mg rutin equivalents per 100 gram of dry weight (mg RE/100 g DW).

### 2.7 Quantification of ascorbic acid content

The content of ascorbic acid was determined by the titration method by 2, 6-dichlorophenol–indophenol with reference to AOAC method No. 967.21 (26). About 2 g of cantaloupe melon samples were homogenized with 30 mL of 1% oxalic acid solution for 30 min. The mixture was then filtered and the supernatant was dilute to 50 mL by the same extracting solvent. 10 mL filtrate were taken and titrated against calibrated 2,6-dichloro-indophenol solution to a pink end point, which lasted for at least 15 s. The volume of the titration solution used was recorded and the same procedure was repeated three times. The results were shown as mg per 100 gram of dry weight (mg/100 g DW).

### 2.8 Quantification of β-carotene content

The extraction of β-carotene was described in 2.6.1. The content of β-carotene in the hexane extract of cantaloupe melon samples was determined by HPLC using a BEH-C18 Symmetry column (150 mm × 2.1 mm, 1.7 μm). The mobile phase was methanol/acetonitrile/tetrahydrofuran (73:20:7, v/v/v) with a flow rate of 1 mL/min and an injection volume of 40 μL at 25°C. The content of β-carotene was determined from peak area. Results were shown as mg per gram of dry weight (mg/g DW) (27).

### 2.9 Quantification of phenolic compounds

The extraction procedure was described in 2.6.3. The content of several phenolic compounds, namely, gallic acid, chlorogenic acid, caffeic acid and rutin were quantified by HPLC using a BEH-C18 Symmetry column (150 mm × 2.1 mm, 1.7 μm) (28). The mobile phase was composed of 0.05% acetic acid in water (A) and acetonitrile (B) with the flow rate of 1 mL/min at 25°C. The eluates were monitored at 280 and 310 nm. The standard compounds of these four phenolic compounds were analyzed under the same analytical conditions. The quantity of these four phenolic compounds were assessed from peak area. Results were shown as mg per 100 gram of dry weight (mg/100 g DW).

### 2.10 Assay of antioxidant capacity

#### 2.10.1 DPPH activity

The DPPH activity was performed with reference to Sanpinit et al. (29). Briefly, 3.9 mL of 0.1 mM DPPH solution was added to 100 μL of extract. The mixture was left at dark for 1 h under room temperature. The absorbance at 517 nm was measured by spectrophotometer (BioTek instruments, Winooski, VT, United States). The results were shown as milligrams of trolox equivalent (TE) per gram of dry weight (mg TE/g DW).

#### 2.10.2 FRAP activity

The FRAP assay was performed as reported by Sanpinit et al. (29). Briefly, 10 mL of 2,4,6-tri(2-pyridyl)- 1,3,5-triazine solution, 10 mL of ferric chloride solution (20 mM), and 100 mL of acetate buffer (300 mM, pH 3.6) were mixed together to form the FRAP reagent. 100 μL of extract was mixed with 3 mL of fresh FRAP reagent and was kept at 37°C in water bath for 4 min. The absorbance at 734 nm was measured by spectrophotometer (BioTek instruments, Winooski, VT, United States). The results were shown as milligrams of trolox equivalent (TE) per gram of dry weight (mg TE/g DW).

#### 2.10.3 ABTS activity

The ABTS activity was performed according to Sanpinit et al. (29). Briefly, 2.6 mM potassium persulfate solution and 7.4 mM ABTS solution was mixed to form the ABTS reagent. The mixture was kept in dark at room temperature for 12 h. And then, it was diluted by distilled water until the absorbance at 734 nm reached 0.700 ± 0.025. Afterwards, 3.0 mL of fresh ABTS reagent was added to 100 μL of extract at proper concentrations. And the mixture was kept in dark at room temperature for another 1 h. The absorbance at 734 nm was measured by spectrophotometer (BioTek instruments, Winooski, VT, United States). The results were shown as milligrams of trolox equivalent (TE) per gram of dry weight (mg TE/g DW).

### 2.11 Assay of polyphenol oxidase activity

#### 2.11.1 Enzyme extraction

About 100 mg of cantaloupe melon samples were mixed with 50 mL of sodium phosphate buffer (pH 6.5, 10 nM) consisting of 1 mol/L NaCl, 0.1 g/100 mL triton X-100, and 4 g/100 mL poly (vinylpyrrolidone). The mixture was grinded thoroughly at 4°C. And then, it was centrifuged at 12,000 × g for 30 min at 4°C. The supernatant was stored at 4°C and the residuals were extracted again as described above. The two supernatants were combined and kept at 4°C and the activity of enzyme was measured shortly (30).

#### 2.11.2 Polyphenol oxidase activity

Three hundred microliter of the enzyme extraction was quickly mixed with 2.7 mL of 0.1 mol/L catechol which was prepared with 0.2 mol/L phosphate buffer at pH 6.5. Afterwards, the increase of absorbance at 420 nm was recorded within 3 min using a spectrophotometer (BioTek Instruments, Winooski, VT, United States). The content of enzyme that leading to the enhance of 0.001 absorbance unit per min was used as the unit of PPO activity. The results were calculated using the following Equation 4:


(4) $$U=\frac{\mathrm{\Delta OD}420\times V}{0.001\times \mathrm{Vs}\times T\times M}$$

Where ΔOD_420_ is the increased value of absorbance within 3 min, V represents the total volume of the PPO extract (mL), *Vs* represents the added volume of PPO extract (mL), T is the reaction time (min), and M represents the mass weight (g).

### 2.12 Statistical analysis

Statistical analysis was done based on three replicates of experiments. The results are shown as the mean ± standard deviation. Statistical differences among different groups were analyzed by SPSS 24.0 (IBM, NY, United States). Differences between the groups were determined by one-way analysis of variance. A *p*-value below than 0.05 was identified as statistically significant.

## 3 Results and discussion

### 3.1 Effect of drying methods on drying time and physical properties of cantaloupe melon slices

#### 3.1.1 Drying time and moisture

As shown in Table 1, different drying methods resulted in significantly different drying time and moisture. The drying time varied from 0.5 to 48 h. Moisture of fresh cantaloupe melon was 90.3%. The highest and the lowest moisture was observed in HD40 (15.2%) and FD (8.5%), respectively.

**Table 1:** Effect of different drying methods on drying time, moisture, rehydration ratio and color of cantaloupe melon slices.

Drying methods	Drying time (h)	Moisture (%, dry weight)	Rehydration ratio (%)	Color			
				L	a	b	ΔE
Fresh	–	90.3 ± 1.2a	–	69.14 ± 0.46a	9.46 ± 0.04a	15.39 ± 0.64f	–
MD	0.5 ± 0.05f	10.2 ± 0.17e	482 ± 4b	54.32 ± 0.37c	7.73 ± 0.07c	21.63 ± 0.98e	16.17 ± 0.26c
FD	48 ± 2a	8.5 ± 0.16e	563 ± 4a	67.64 ± 0.85b	8.14 ± 0.12b	20.54 ± 1.32d	5.52 ± 0.97d
HD40	10.5 ± 0.5b	15.2 ± 0.23b	436 ± 3d	50.78 ± 1.64d	7.06 ± 0.15c	24.06 ± 1.24d	20.45 ± 0.75b
HD50	8 ± 0.5c	14.2 ± 0.36c	443 ± 4d	46.54 ± 0.62e	7.98 ± 0.11c	26.63 ± 0.93c	25.28 ± 0.57a
HD60	7.5 ± 0.5d	11.7 ± 0.27d	464 ± 4c	51.39 ± 0.71d	7.51 ± 0.08d	27.64 ± 0.87b	21.65 ± 0.96b
HD70	6 ± 0.2e	12.2 ± 0.26d	452 ± 6 cd	47.26 ± 1.23e	6.35 ± 0.09e	29.38 ± 0.92a	26.16 ± 0.53a

Microwave drying (MD); Freeze drying (FD); Hot air drying at 40°C (HD40), 50°C (HD50), 60°C (HD60), and 70°C (HD70). Different letters in the same column indicate significant differences between treatments, *p* < 0.05.

#### 3.1.2 Color

Color is an important indicator of food quality, which can effectively improve consumers’ acceptance of products (31). It can be seen from Table 1 that the L*, a*, b*, and ΔE values of cantaloupe melon slices after each drying method were significantly different (*p* < 0.05). The lightness (L*) of the cantaloupe melon was 69.14, the redness (a*) was 9.46, and the yellowness (b*) was 15.39. Compared with fresh cantaloupe melon, L*value of the dry cantaloupe melon slices in FD, MD, HD60, HD40, HD70, and HD50 decreased by 2.17, 21.43, 25.67, 26.55, 31.65, and 32.69%, respectively. And this decreased trend was also found in a* value. While all drying methods increased the value of yellowness (b*). As shown in Table 1, the highest a* value of dry cantaloupe melon slices was found in FD treatment (8.14) and the lowest was in HD70 (6.35). ΔE is the combination of L*, a*, b*, and has been widely used to characterize the variation of colors in food samples after processing. Compared with other treatments, the change of ΔE was the smallest in FD (5.52). Overall, FD treated samples had better color retention, followed by MD (16.17), HD40 (20.45), and HD60 (21.65), while the color of HD70 (26.16) was the worst. The color changes in cantaloupe melon slices caused by thermal process may be not only because of the non-enzymatic browning reaction, but also because of the destruction of pigments present in the cantaloupe melon (32). In addition, relative humidity, temperature, and drying time during the drying process also affect the color of dry cantaloupe melon slices (4, 32).

#### 3.1.3 Volume shrinkage ratio and hardness

**Figure 1:**
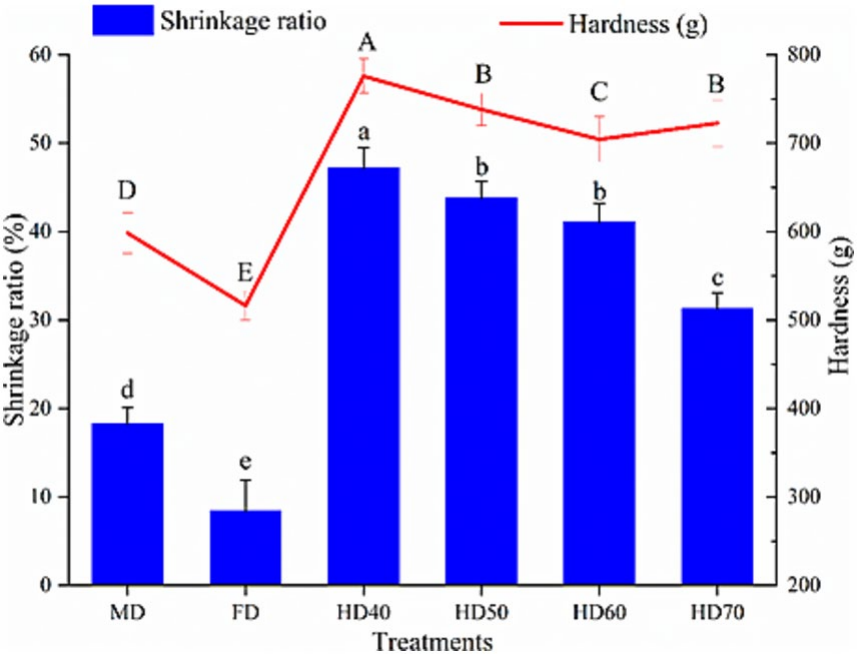
Effect of different drying methods on volume shrinkage ratio and hardness of cantaloupe melon slices. Microwave drying (MD); Freeze drying (FD); Hot air drying at 40°C (HD40), 50°C (HD50), 60°C (HD60), and 70°C (HD70). Different letters indicate significant differences between treatments, *p* < 0.05.

Dehydration can cause changes in the volume (expansion or shrinkage) along with water loss of fruits and vegetables. Volume shrinkage ratio of cantaloupe melon slices dehydrated by different drying methods were presented in Figure 1. As can be seen, all dying treatments caused significant shrinkage, with the lowest volume shrinkage ratio in FD (8.42%) and the highest in HD40 (47.23%). Moderate volume shrinkage ratio (18.71%) was observed in cantaloupe melon slices treated by MD. It has also been reported that freeze drying induced the minimum volume shrinkage ratio while the hot air drying or oven drying lead to the highest volume shrinkage ratio in roses (33) and garlic slices (34). The significant volume shrinkage as a result of heat drying is because of the evaporation of the surface water, resulting in a difference in pressure between the inside and outside of fruits, thus leading to obvious changes in the size and shape of fruits (34). FD treatment caused the minimum volume shrinkage ratio, therefore maintaining the best structural integrity of cantaloupe melon slices. This was probably ascribed to the lower sublimation of ice from frozen samples in FD (35). MD also caused moderate shrinkage ratio, which might be caused by absorbing microwave energy, thus accelerating the water evaporation from the matrix and leading to tissue collapse (36). The hardness values of dehydrated cantaloupe melon slices dried by different treatments are presented in Figure 1. It was found that FD dried cantaloupe melon slices possessed the lowest hardness (516.32 g), followed by MD (598.43 g), HD60 (704.46 g), HD70 (722.69 g), HD50 (738.27 g), and HD40 (775.86 g). The highest hardness in HD was caused by the significant shrinkage in the HD process. A similar result that hot air drying lead to a higher hardness value than MD and FD was found in dried cabbage (37) and garlic (34). It has been demonstrated that the hardness of dried food was positively connected to the shrinkage ratio in drying (34).

#### 3.1.4 Rehydration ratio

For dried plant-based food, rehydration ratio is frequently used as a characteristic of structural quality, which largely depend on the drying methods employed. Table 1 shows the rehydration ratios of dehydrated cantaloupe melon slices under different drying methods. The rehydration ratios, listed from high to low for FD, MD, HD60, HD70, HD50, and HD40 were 563, 482, 464, 452, 443, and 436%, respectively. FD and MD dried cantaloupe melon slices exhibited the highest rehydration capacity than hot air drying methods. Among the different temperatures of HD, HD60 lead to high rehydration rate. The rehydration process depends on the structure between cells and tissues that are changed by drying. A highly porous structure is usually considered as an ideal structure for an effective rehydration, as it allows high water absorbance (37). Sublimation of water from the samples under FD treatment can facilitate the samples to retain their porous structure, which promoted the rehydration capacity of the FD dried cantaloupe melon slices. In comparison, HD caused the serious shrinkage and dense structure of cantaloupe melon slices, possibly leading to collapsed capillaries, thereby resulting in a relatively low rehydration capacity. Consistent with our findings, previous studies have shown that FD and MD dried cabbages showed higher rehydration ratio than other methods (37).

### 3.2 Effect of drying methods on the total amino acids content and total sugar content of cantaloupe melon slices

**Figure 2:**
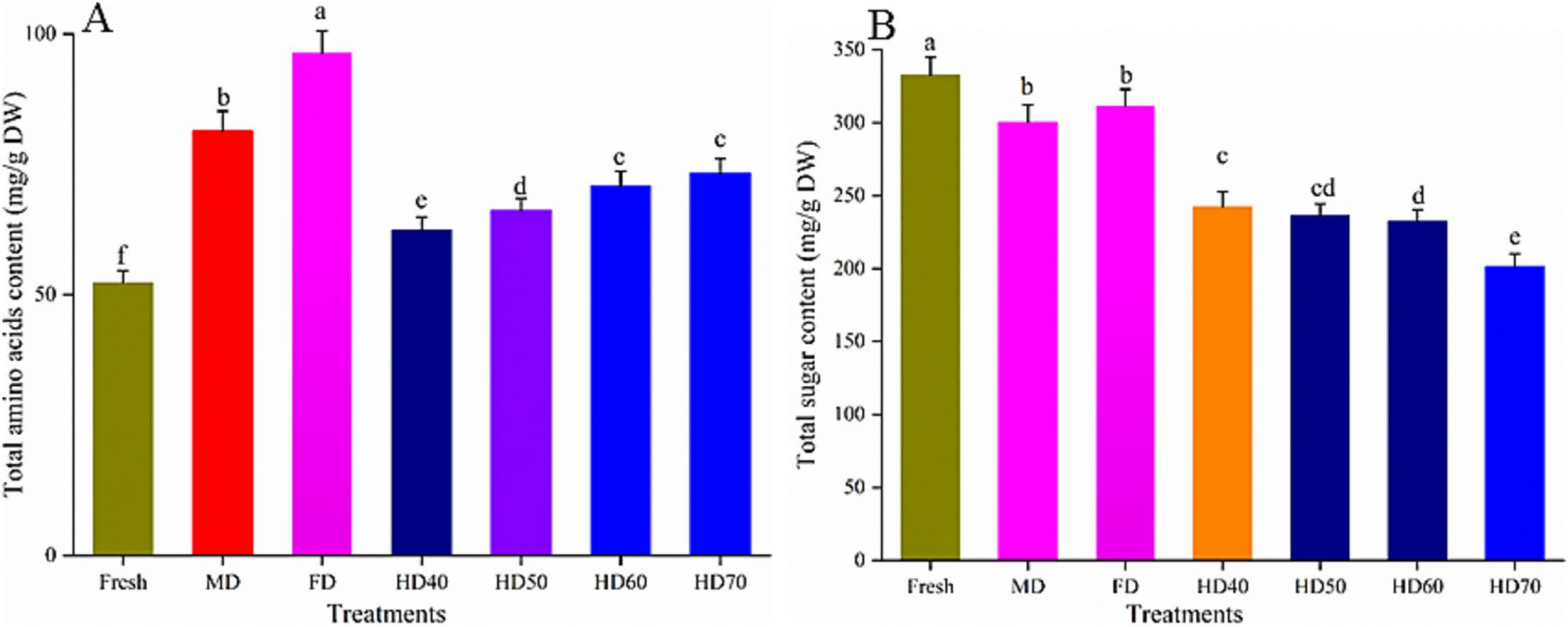
Effect of different drying methods on total amino acids content **(A)** and total sugar content **(B)** of cantaloupe melon slices. Microwave drying (MD); Freeze drying (FD); Hot air drying at 40°C (HD40), 50°C (HD50), 60°C (HD60), and 70°C (HD70). Different letters indicate significant differences between treatments, *p* < 0.05.

As shown in Figure 2A, after all drying treatments, total amino acids content (TAC) in cantaloupe melon slices was significantly higher than that in fresh cantaloupe melon (52.36 mg/g DW). The TAC reached a maximum value of 96.43 mg/g DW after FD treatment. The TAC in MD, HD70, HD60, HD50, and HD40 was 81.42, 73.31, 70.85, 66.27, and 62.49 mg/g DW, respectively. The increasing effect was more significant when the drying temperature was higher. However, the TAC between HD60 and HD70 was not significant (*p* > 0.5). It has been reported that the content of TAC in cantaloupe melon was 4.5–7.2 mg/g fresh weight (38), and 8.42 mg/g fresh weight (39). Taking into account the moisture content of over 90% in melons, the amino acid content obtained in the present study agreed with previous studies (38, 39). Similar trends have also been reported that the total amino acids content of 31.70 mg/g DW in fresh mushrooms was increased to 60.02 mg/g DW by hot air drying (40). Compared with fresh mushrooms, freeze drying and vacuum drying caused higher total free amino acids than HD and MD (40). And the increase trend was more significant with relative higher temperature. This phenomenon maybe caused by protein degradation induced by high temperature. Another explanation could be that under low temperature (HD40) drying condition, the cells were still alive with strong respiration, metabolism, and consumption of organic matters and energy, and finally lead to the loss of nutritious components. However, there were also some contrary reports (40). In green seaweed samples, freeze drying caused the highest loss of TAC (31.9%), followed by solar drying (24.6%) and vacuum drying (13.1%) (22). This maybe because the specific composition of seaweed was more susceptible to drying. It has been summarized that the main factors causing variations in TAC during drying process were as follows. Firstly, amino acids are released out increasingly with time and temperature. Secondly, a relative higher temperature may lead more amino acids released because of protein degradation slowly. Thirdly, Maillard reaction related to free amino acids can cause the loss of amino acids (41).

As shown in Figure 2B, compared with fresh cantaloupe melon, all drying methods lead to the loss of total sugar content (TSC) of cantaloupe melon slices. The TSC decreased with the following order: fresh (332.63 mg/g DW), FD (311.71 mg/g DW, 6.28% loss), MD (300.4 mg/g DW, 9.67% loss), HD40 (242.35 mg/g DW, 27.15% loss), HD50 (236.24 mg/g DW, 28.98% loss), HD60 (232.42 mg/g DW, 30.13% loss), and HD70 (201.31 mg/g DW, 39.48% loss). FD and MD treatments had better effect in retaining sugar content. Higher temperature caused more loss of TSC. However, the loss caused by HD50 and HD60 was not significant (*p* > 0.5). It has also been reported that freeze drying caused the least loss of sugar content in sweet corn (42). This may because lower temperature can decrease some related enzymatic and chemical reactions, which lead to the reduced loss of sugar (42). The relatively shorter drying time and lower temperature in MD lead to the minor influence on the loss of the sugar. Thus, the retention rate of TSC under MD was also higher than that under HD. Drying conditions significantly affect the technological and functional characteristics of carbohydrates (43). Similar to our results, Miranda et al. (44) found that all drying temperatures caused a decrease in the sucrose content of quinoa seeds, with the greatest loss of 56% in high drying temperature (80°C) (25). Xu et al. (40) also demonstrated that total sugar content of mushroom gradually decreased with increased drying temperature.

### 3.3 Effect of drying methods on the total phenolics content, total flavonoids content and total carotenoids content of cantaloupe melon slices

**Figure 3:**
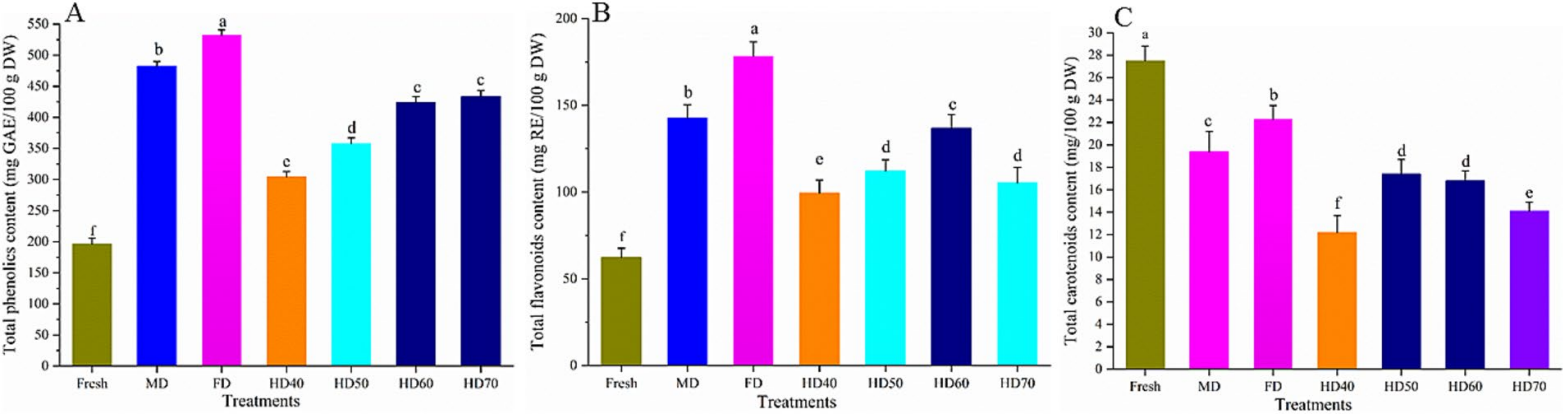
Effect of different drying methods on total phenolics content **(A)**, total flavonoids content **(B)** and total carotenoids content **(C)** of cantaloupe melon slices. Microwave drying (MD); Freeze drying (FD); Hot air drying at 40°C (HD40), 50°C (HD50), 60°C (HD60), and 70°C (HD70). Different letters indicate significant differences between treatments, *p* < 0.05.

As shown in Figure 3, the total phenolics content (TPC) (Figure 3A), total flavonoids content (TFC) (Figure 3B), and total carotenoids content (TCC) (Figure 3C) varied significantly among different drying methods. Compared with fresh cantaloupe melon, all drying methods applied caused a significant (*p* < 0.05) increase in TPC and TFC and a significant (*p* < 0.05) decrease in TCC. The TPC of the fresh cantaloupe melon was 196.35 mg GAE/100 g DW, while that in FD, MD, HD70, HD60, HD50, and HD40 was 532.61, 482.36, 433.62, 424.63, 357.87, 304.31 mg GAE/100 g DW, respectively. The TFC of the fresh cantaloupe melon was 62.54 mg RE/100 g DW. It is also the FD treatment that caused the most significant increase in TFC (178.42 mg RE/100 g DW, 2.86 fold), followed by MD (142.64 mg RE/g DW, 2.29 fold), HD60 (136.85 mg RE/100 g DW, 2.19 fold), HD50 (112.33 mg RE/100 g DW, 1.8 fold), and HD40 (99.46 mg RE/100 g DW, 1.59 fold). From these results, it can be clearly seen that FD increased the TPC and TFC most significantly. Of the hot air drying treatments, HD60 showed the best effect in increasing TPC and TFC.

These results demonstrated that phenols and flavonoids were easier to be extracted from dried cantaloupe melon. This is probably because the structure of cantaloupe melon was changed during the drying process, which may facilitate the release of phytochemicals during homogenization and extraction process (25). It has been suggested that ice crystals formed in FD will push and compress the cells, which will finally cause the release of some bound phenolic components from cells (45). Some previous researches have demonstrated that when MD energy is used for drying, it is instantly absorbed and converted into heat, and increased the temperature of the sample. Subsequently, water is vapored due to the sudden enhance in pressure of the sample. Thus, the bound phenolic components are released (7). Besides, the chemical structures of some phenolic compounds dissociated by microwave drying can be altered and turned into more soluble forms, making the phenolic compounds easier for quantification (46). As for the reasons the increasing effect was less in HD, one explanation is that water is firstly evaporated from the surface of sample in HD, leading to sclerosis and cell collapse, which inhibited the release of phenolics to some extent (46).

As for TCC, the highest value was in fresh cantaloupe melon (27.58 mg/100 g DW), with the descending order of FD (22.35 mg/100 g DW), MD (19.42 mg/100 g DW), HD50 (17.42 mg/100 g DW), HD60 (16.81 mg/100 g DW), HD70 (14.17 mg/100 g DW) and HD40 (12.23 mg/100 g DW). FD had the best effect in retaining TCC and MD was better than HD. Of the hot air drying treatments, HD50 and HD60 showed better effect in retaining TCC than others, with no significant difference between them (*p* > 0.05). One of the main reasons for the loss of carotenoids in the current study was the exposure of carotenoids to light, heat, and oxygen after the carotenoids released (47). The activities of related enzymes causing the degradation of carotenoids may also lead to the decrease of carotenoids. The low temperature and oxygen-free environment in FD might help to minimize the degradation of carotenoids. It has been demonstrated that the optimal range for the carotenoids degraded enzymes was 30–45°C and the longer drying time of HD40 lead to the more loss of carotenoids (48).

It has been widely reported that food drying methods can influence the release of phytochemicals in fruits due to microstructural changes. And the changes may lead to negative, neutral or positive effects in phytochemicals. Several factors such as drying methods, type of phytochemicals, and their intracellular localization may influence the change (43). Some previous studies showed that drying is very effective for increasing the TPC and TFC in different food items, such as dried tomatoes (49), Soursop leaves (50), apricots (51) and raisins (52). However, some other researches (53) reported that TPC and TFC decreased during thermal process, others (54) have reported no significant changes. It has also been reported that freeze drying can be the most appropriate method for preserving carotenoids (55), while there were also some other researches demonstrating that few or no differences among different drying methods in preserving carotenoids (56). These contrasting data, as well as our results, suggest that the food matrices that containing these phytochemicals are important in considering the influences of drying methods.

### 3.4 Effect of drying methods on the content of individual bioactive compounds of cantaloupe melon slices

**Figure 4:**
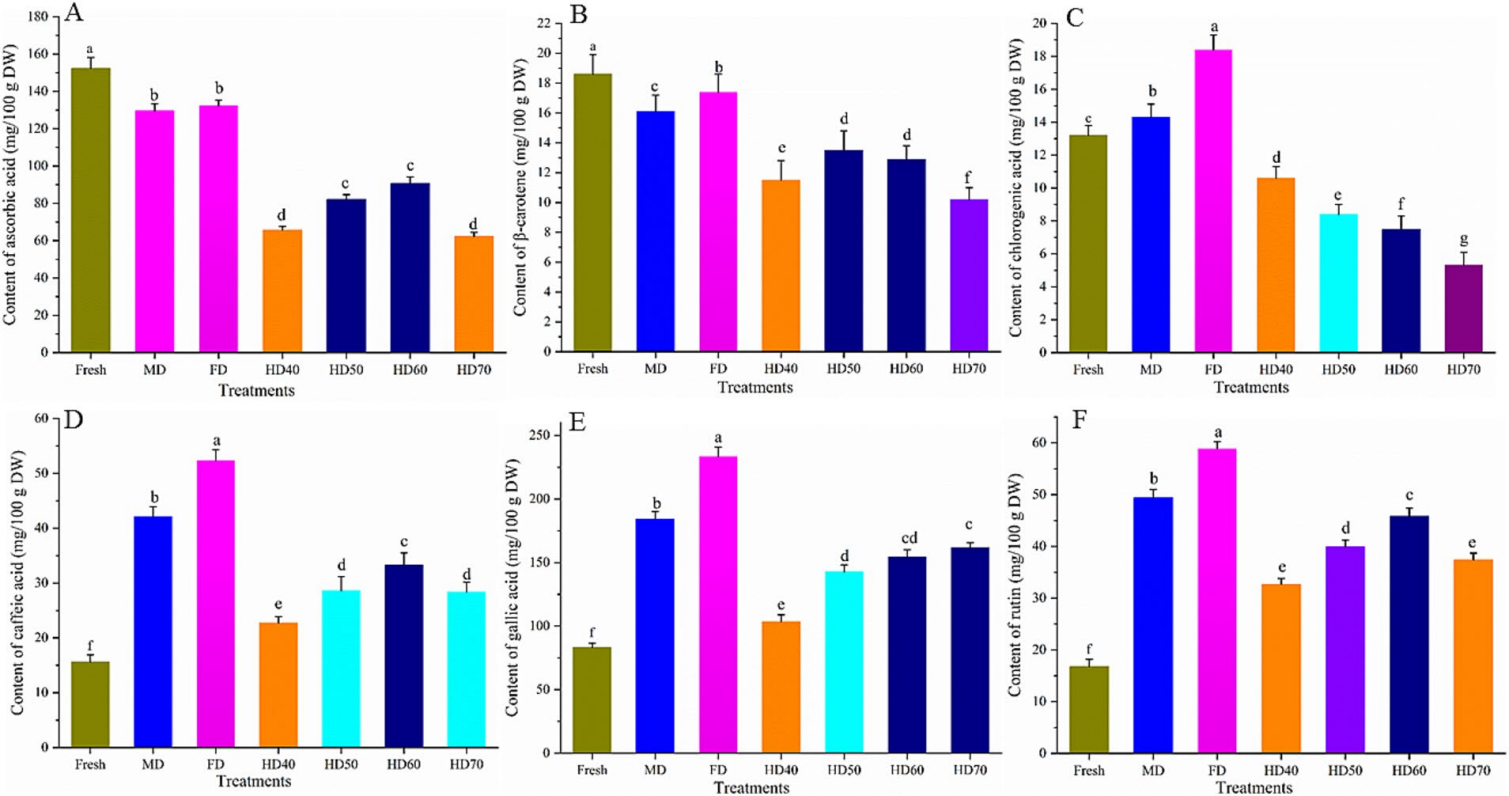
Effect of different drying methods on the content of ascorbic acid **(A)**, β-carotene **(B)**, chlorogenic acid **(C)**, caffeic acid **(D)**, gallic acid **(E)** and rutin **(F)** of cantaloupe melon slices. Microwave drying (MD); Freeze drying (FD); Hot air drying at 40°C (HD40), 50°C (HD50), 60°C (HD60), and 70°C (HD70). Different letters indicate significant differences between treatments, *p* < 0.05.

The content of six individual bioactive compounds, namely, ascorbic acid, β-carotene, rutin, gallic acid, chlorogenic acid, and caffeic acid as affected by different drying methods were evaluated. The results are shown in Figure 4.

The content of ascorbic acid (Figure 4A) and β-carotene (Figure 4B) was significantly decreased after drying. In the fresh cantaloupe melon, ascorbic acid content was 153.3 mg/100 g DW. The highest ascorbic acid in dried cantaloupe melon slices was obtained in freeze drying (132.42 mg/100 g DW), followed by MD (129.55 mg/100 g DW), HD60 (90.85 mg/100 g DW), HD50 (82.2 mg/100 g DW), HD40 (65.73 mg/100 g DW), and HD70 (62.32 mg/100 g DW). As for β-carotene, HD70 had the least β-carotene content with the loss of 45.16%. There was no significant difference between the loss of HD50 (27.42%) and HD60 (30.65%). FD caused the least loss of 6.45%. MD caused a moderate loss of 13.45%.

The quantity of ascorbic acid lost in vegetables and fruits during drying depends on the physical properties of the product and the type of process (57). Ascorbic acid was oxidized and its content was decreased in high temperatures and long drying times (57). The amount of ascorbic acid in MD was significantly higher than that in HD. This might be due to the shorter drying time in MD. Previous studies also illustrated that shorter drying time and lower temperature could maintain ascorbic acid (58). In agreement with our study, it was reported that β-carotene content in pumpkin slices was the lowest in HD and the highest in FD (59). The highest loss of β-carotene has also been verified in dried pepper subjected to sun drying at higher temperatures (60).

The content of chlorogenic acid (Figure 4C) under FD, MD, HD40, HD50, HD50, and HD70 was 18.42, 14.31, 10.63, 8.41, 7.52, and 5.33 mg/100 g DW. Compared with that in fresh cantaloupe melon (13.2 mg/100 g DW), the content of chlorogenic acid increased significantly (*p* < 0.05) in FD and MD, whereas hot air drying caused a significant decrease in the content of chlorogenic acid. Chlorogenic acid is a polyphenolic compound sensitive to thermal treatment and oxygen (61). It has been reported that the increase of oven temperature (55–75°C) caused a significant decrease of chlorogenic acid in chrysanthemum flower. The increase of chlorogenic acid in FD might be due to the drying conditions (low temperature, low pressure, non ionizing electromagnetic energy), which inhibit the activity of polyphenol oxidases, thus leading to a relatively higher content of chlorogenic acid in the dried samples (61).

Freeze drying treatments caused a significant increase of the content of caffeic acid (Figure 4D), gallic acid (Figure 4E) and rutin (Figure 4F). Caffeic acid can be detected in fresh cantaloupe melon, FD, MD, HD60, HD50, HD70, and HD40 with the content of 15.6, 42.13, 52.41, 33.34, 28.66, 28.42, and 22.75 mg/100 g DW, respectively. Gallic acid content of cantaloupe melon ranged from 83.24 mg/100 g (fresh sample) to 233.61 mg/100 g (FD sample). Of the hot air treatment, the highest gallic acid content was found in HD60 (154.37 mg/100 g) and HD70 (161.71 mg/100 g), and there was no significant difference between them. The increase rate of rutin was minimal in HD40 (1.95 times) and HD70 (2.23 times) and was the largest in FD (3.51 times).

Lemus-Mondaca et al. (28) reported that all drying methods lead to a significant (*p* < 0.05) increase in caffeic acid compared with fresh *Stevia rebaudiana* leaves. On the contrary, caffeic acid and some of its derivatives were lost in *Echinacea Purpurea* after HD at 55°C and 70°C (62). Consistent with our research, gallic acid content in ginger rhizomes was reported to be increased under freeze drying, microwave oven drying, oven drying and air drying with the greatest increase in FD treatment (63). Siriamornpun et al. (64) also reported that thermal drying resulted in higher contents of rutin in marigold flower. Concentration of rutin was also reported to be significantly higher in dried leaves of Nightshade than that in fresh samples (65). However, there are also some reports demonstrating that drying caused a significant loss of rutin in *Stevia rebaudiana* leaves, and the loss reached 72.42% in the infrared drying samples (28).

### 3.5 Effect of drying methods on the antioxidant activities of cantaloupe melon slices

**Figure 5:**
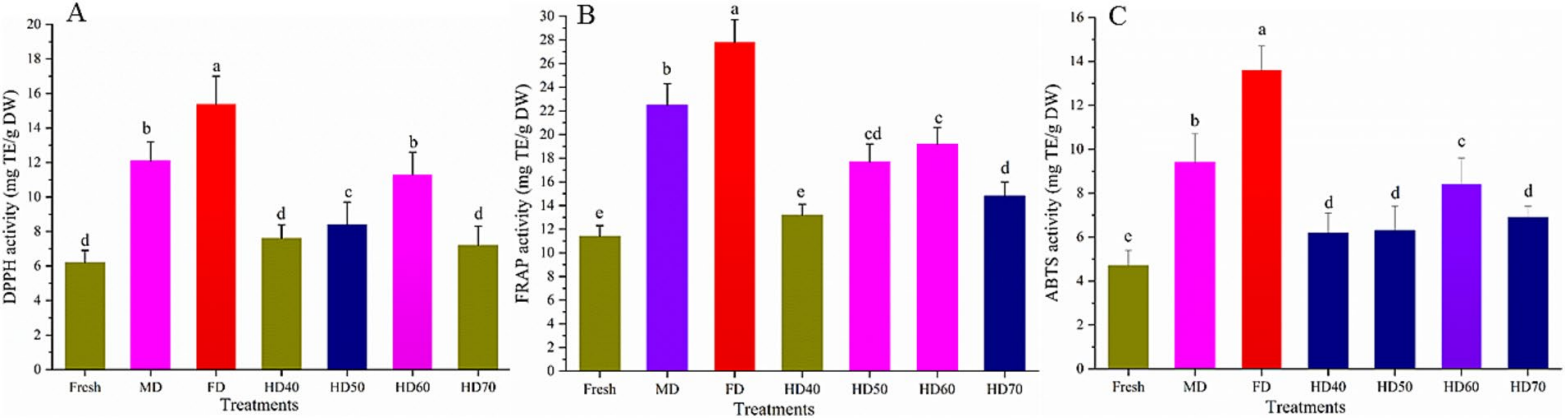
Effect of different drying methods on the antioxidant activities of cantaloupe melon slices. **(A)** DPPH, **(B)** FRAP and **(C)** ABTS. Microwave drying (MD); Freeze drying (FD); Hot air drying at 40°C (HD40), 50°C (HD50), 60°C (HD60), and 70°C (HD70). Different letters indicate significant differences between treatments, *p* < 0.05.

The DPPH and ABTS radical scavenging activity methods are on account of the single electron transfer ability from antioxidant for DPPH or ABTS free radical in forming an electron pair. The reaction causes a lighter color of the solution. The stable free radicals, namely, DPPH and ferric ion, were as used as oxidants for DPPH and ABTS method, respectively. The ferric reducing antioxidant power (FRAP) method is in accordance with the ferric reduction or copper ion ability, which causes a darker color of the solution. As each assay of antioxidant capacity evaluates a restricted level of antioxidant ability, it is needed to apply different methods in evaluating the antioxidant capacity of phytochemicals (66). In the current study, the antioxidant ability of fresh and dried cantaloupe melon as influenced by different drying methods was assessed by the three commonly used methods mentioned above. The results are shown in Figure 5.

Compared with fresh cantaloupe melon, all drying methods used caused a significant increase (*p* < 0.05) in antioxidant activity. The DPPH activity (Figure 5A) of ethanolic extracts of cantaloupe melon ranged from 6.27 to 15.41 mg TE/g DW, while that of FRAP (Figure 5B) and ABTS (Figure 5C) were from 11.43 to 27.82 mg TE/g DW, and 4.73 to 13.62 mg TE/g DW, respectively. These results agreed with previous reports that cantaloupe melon possessed high levels of antioxidant capacity (67). DPPH of cantaloupe melon was the highest in freeze drying (15.41 mg TE/g DW), followed by MD (12.13 mg TE/g DW), HD60 (11.39 mg TE/g DW), HD50 (8.42 mg TE/g DW), HD40 (7.61 mg TE/g DW), HD70 (7.24 mg TE/g DW), and fresh (6.25 mg TE/g DW). No significant difference in DPPH was observed between MD and HD60. Although HD40 and HD70 treated cantaloupe melon slices showed higher DPPH activity than fresh one, there was no significant difference between them. In terms of FRAP activity, the highest value was found at FD (27.82 mg TE/g DW), a moderate value at MD (22.51 mg TE/g DW), HD60 (19.23 mg TE/g DW), HD50 (17.71 mg TE/g DW) and HD70 (14.81 mg TE/g DW), while the lowest was found in HD40 (13.28 mg TE/g DW) and fresh (11.45 mg TE/g DW). With regard to the ABTS values, it is observed that the antioxidant capacity was the greatest in FD (13.61 mg TE/g DW), with the descending order of MD (9.44 mg TE/g DW), HD60 (8.46 mg TE/g DW), HD70 (6.91 mg TE/g DW), HD50 (6.31 mg TE/g DW), HD40 (6.23 mg TE/g DW) and fresh (4.71 mg TE/g DW). There was no significant difference between HD70, HD50 and HD40 treated samples.

These results clearly indicated that drying methods significantly influenced the antioxidant properties of cantaloupe melon. In general, FD treatment resulted in the highest antioxidant capacity of cantaloupe melon in all three assays. Therefore, FD can be used to dry some high quality and high valued cantaloupe melon. MD treatment had higher antioxidant activity than HD. 60°C was suggested for drying the cantaloupe melon based on hot air drying method. It has been reported that the antioxidant capacity of plants would be significantly increased after fresh plants were dried (68), and similar phenomenon was also observed in this study. It has been also reported that FD was the most appropriate drying method with regard to the antioxidant activity of chokeberry, mulberry, Chinese ginger and kiwi fruit (69, 70). It was indicated that microwave radiation may lead to the break of covalent bonds between phytochemicals to release and activate natural antioxidants (71). Microwave could also inhibit the activity of oxidative and hydrolytic enzymes to restrain the loss of antioxidants (69). The relatively shorter drying time used in HD60 may help to keep certain compounds responsible for the greater antioxidant activities compared with HD40 and HD50. This agrees with the report that antioxidant activity is not only connected with the content and profile of phenolic compounds, but also with the presence of other phytochemicals released from the matrix that exhibit reducing activity (72). Although it took shorter time in HD70 than HD60, some active compounds may degrade in HD70, resulting in the lower antioxidant activity of HD70. The results that freeze drying treatment showed higher scavenging activity has also been found in *Hibiscus sabdariffa* L. (73), rose flower (74), marigold (64), and tribe Genisteae (*Fabaceae*) (75). However, there are also some contradictory findings. Periche et al. (45) found that the highest antioxidant activity of CD Stevia was observed under 180°C and the relatively lower antioxidant activity was found in freeze drying and shade drying extracts. The conflicting results may be ascribed to the different active compounds that are responsible for the antioxidant activity in different plant species.

### 3.6 Effect of drying methods on the polyphenol oxidase activities of cantaloupe melon slices

**Figure 6:**
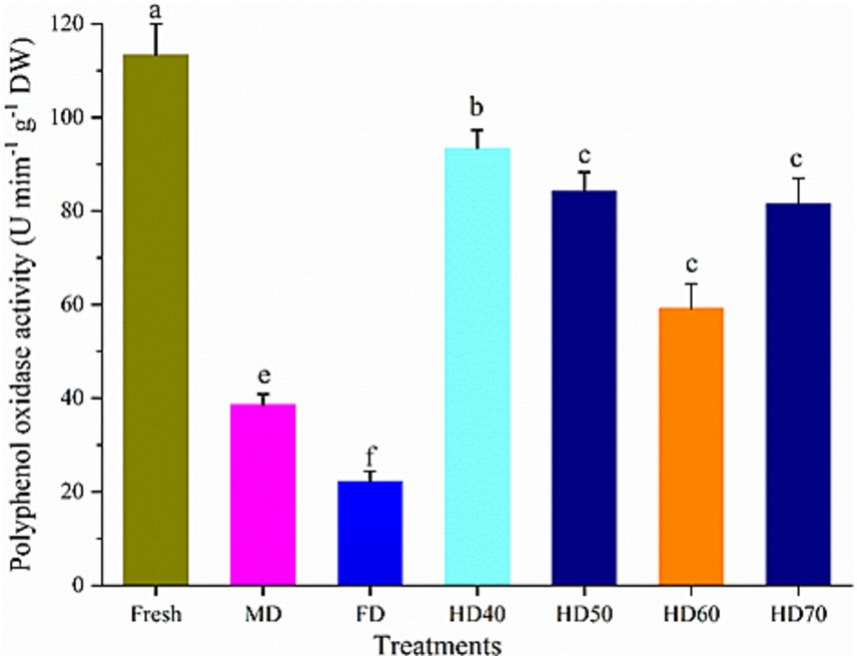
Effect of different drying methods on the polyphenol oxidase activities of cantaloupe melon slices. Microwave drying (MD); Freeze drying (FD); Hot air drying at 40°C (HD40), 50°C (HD50), 60°C (HD60), and 70°C (HD70). Different letters indicate significant differences between treatments, *p* < 0.05.

Enzymatic browning is an important issue in fresh fruits, often leading to negative effects on color, flavor and taste. This reaction is mainly because of the oxidation of polyphenol compounds by polyphenol oxidase (PPO). PPO is an oxidoreductase that catalyzes the hydroxylation of monophenols to diphenols, which is further oxidized to orthoquinone (76). This can cause natural antioxidants suffered from rapid enzymatic oxidation. The activities of PPO in dried cantaloupe melon slices may explain the increase of their antioxidant activities. Figure 6 shows the PPO activity in fresh and dried cantaloupe melon subjected to different drying methods. Different drying methods lead to significant differences in PPO activity. The highest PPO activity was found in fresh cantaloupe melon (113.35 U mim^−1^ g^−1^ DW), followed by HD40 (93.41 U mim^−1^ g^−1^ DW), HD50 (84.23 U mim^−1^ g^−1^ DW), HD70 (81.52 U mim^−1^ g^−1^ DW), MD (38.67 U mim^−1^ g^−1^ DW), and FD (22.37 mim^−1^ g^−1^ DW) methods. In our study, compared with dried cantaloupe melon slices, the lowest content of TPC, TFC and antioxidant activities were found in fresh samples. This phenomenon was probably because of the inhibition of related enzymes such as PPO during the procedure. It has been reported that the activity of PPO was fully inhibited by drying, and the relatively lower TPC and TFC in fresh samples were because of the oxidizing by PPO (77). It has also been demonstrated that inhibition the activity of PPO may lead to the dried plants had more secondary metabolites than fresh ones (78).

## 4 Conclusion

Three different drying methods have been compared for their influences on the quality, content of bioactive compounds, and antioxidant activities of cantaloupe melon slices. Drying methods included microwave drying (MD), freeze drying (FD), and hot air drying at 40°C (HD40), 50°C (HD50), 60°C (HD60), and 70°C (HD70). According to the results of present study, we concluded that drying methods and temperature had significant influences on the quality, active ingredients, and antioxidant activities of the dehydrated cantaloupe melon slices. Compared with MD and HD, FD resulted in a lower volume shrinkage and hardness, higher retention of color, more active components, phenolic compounds profiles, higher antioxidant activity, and lower polyphenol oxidase activity, which was possibly attributed to relatively less intense heating during FD treatment. However, it is only practicable for drying high valued cantaloupe melon slices because of the high cost of FD. The quality and antioxidant activity of MD was better than HD. Of the HD treatments, HD60 was better than others. Considering the cost of HD and the actual conditions, MD and HD60 was also suggested as a practically alternative method for commercial scale production of cantaloupe melon slices with relatively higher quality and functions.

## Data Availability

The datasets presented in this study can be found in online repositories. The names of the repository/repositories and accession number(s) can be found in the article/supplementary material.
